# The multiscale backbone of the human phenotype network based on biological pathways

**DOI:** 10.1186/1756-0381-7-1

**Published:** 2014-01-25

**Authors:** Christian Darabos, Marquitta J White, Britney E Graham, Derek N Leung, Scott M Williams, Jason H Moore

**Affiliations:** 1 Department of Genetics, Institute for Quantitative Biomedical Sciences, Dartmouth College, Hanover, NH, USA; 2 Center for Human Genetics Research, Vanderbilt University, Nashville, TN, USA

**Keywords:** Diseasome, Phenotypes, GWAS, Network, Information theory, Biological pathways

## Abstract

**Background:**

Networks are commonly used to represent and analyze large and complex systems of interacting elements. In systems biology, human disease networks show interactions between disorders sharing common genetic background. We built pathway-based human phenotype network (PHPN) of over 800 physical attributes, diseases, and behavioral traits; based on about 2,300 genes and 1,200 biological pathways. Using GWAS phenotype-to-genes associations, and pathway data from Reactome, we connect human traits based on the common patterns of human biological pathways, detecting more pleiotropic effects, and expanding previous studies from a gene-centric approach to that of shared cell-processes.

**Results:**

The resulting network has a heavily right-skewed degree distribution, placing it in the *scale-free* region of the network topologies spectrum. We extract the *multi-scale information backbone* of the PHPN based on the local densities of the network and discarding weak connection. Using a standard community detection algorithm, we construct *phenotype modules* of similar traits without applying expert biological knowledge. These modules can be assimilated to the disease classes. However, we are able to classify phenotypes according to shared biology, and not arbitrary disease classes. We present examples of expected clinical connections identified by PHPN as proof of principle.

**Conclusions:**

We unveil a previously uncharacterized connection between phenotype modules and discuss potential mechanistic connections that are obvious only in retrospect. The PHPN shows tremendous potential to become a useful tool both in the unveiling of the diseases’ common biology, and in the elaboration of diagnosis and treatments.

## Introduction

In this age of system-wide biology, in which organisms and their environment are considered as a whole, a new field has emerged; studying diseases in relationship to one another. Pioneering studies, such as Goh *et al.*’s [1] have resulted in the definition of the Human Disease Network (HDN). Elucidating relationships between human traits or diseases is becoming increasingly - genetic disorders. These traits may be related through shared genes, proteins, or regulatory elements, and identifying commonalities that may reveal shared biological mechanisms. Ultimately, a thorough understanding of these connections will provide tools necessary to design drug targets. The potential for increased biological understanding, and the future clinical impact, justifies the creation of methods optimized to explore the full phenotype genotype spectrum. Genome-wide association studies (GWAS) have helped identify genetic and environmental variants that affect susceptibility to human disease using an agnostic, or hypothesis-free, approach. Such studies offer the promise of personalized diagnostics, prognostics, and medical treatments [2]. Moreover, they provide us with an unprecedented ability to study the genetic interactions between seemingly unrelated traits. To date, approximately 6,000 single nucleotide polymorphisms (SNPs) have been reported as genetic risk-variants for 800+ diseases and traits. Combining data from hundreds of GWASs, takes advantage of the information gained about genotype-phenotype relationships beyond the scope of any single study. This approach provides a novel perspective to integrate genetic, cellular, physiological and clinical data to elucidate the pathobiology of many traits. To this end, modern computational methods, utilizing integrated modeling, are critical as these methods can tackle unprecedented volumes of data. Computational methods are, however, only as good as their translatability into usable observations. Because of the sheer complexity and the number of phenotype-interactions, the usable models need to be intuitive and scalable and have the ability to filter “irrelevant” information, and highlight commonalities between phenotypes. Modeling complex biological systems using network analysis offers a promising approach to evaluate the macro-relationships between these biological components, particularly between certain phenotypes and diseases. Indeed, networks offer relatively straightforward and intuitive representations of interaction phenomena, and allow sophisticated statistical analysis of their intrinsic properties. In addition, methods derived from information sciences and social sciences have proven to leverage the network topology as a source of knowledge, offering sophisticated filtering and grouping techniques. Moreover, these methods work regardless of the actual underlying data type and are therefore applicable to complex networks of phenotypes.

We present the Pathway-based Human Phenotype Network (PHPN), a biological pathway-based mathematical model of a network of human phenotypic traits (PT) visually represented as graphs. Previous network-based studies of diseases have proven useful for envisaging large disease datasets grouped by common genes, similar gene expression profiles, or shared protein interactions [1,3,4]. However, a gene-centric focus has biased the generation and interpretation of these networks, as coding regions constitute less than 2% of the human genome. In a previous study [5], we focused our efforts on constructing sets of risk-associated single nucleotide polymorphisms (SNPs) and SNP bins in linkage disequilibrium to find commonalities between PTs; this SNP clustering approach successfully overcame some weaknesses of previous gene-centric models. In the present article, we describe a method of building a PHPN relying on biological pathways rather than genes. We link PTs that have shared pathways, using mapped genes from GWAS data and gene-to-pathway associations from Reactome, a curated pathway database. Furthermore, we extract the *information backbone* using Serrano *et al.*’s disparity filter [6], to capture the most relevant information from the extremely dense network that results from raw data. Finally, we classify each PT in the network and build *communities* or modules of PTs that are strongly linked, and therefore, show evidence of pleiotropic effects and shared biology. These groups are formed independently of the actual disease classes, based solely on intrinsic network properties.

## Background

In this section, we define the fundamental concepts used in the Methods section below to build the PHPN.

### Genetic data

The catalog of published GWAS maintained by the National Human Genome Research Institute (NHGRI) at the National Institute of Health aggregates studies that report phenotype-to-SNP(s) and phenotype/SNP-to-gene associations (http://www.genome.gov/gwastudies/). The NHGRI catalog, downloaded in March 2013, was the primary source of PT-to-gene to association data. It reports over 800 PTs associated with approximately 2,300 genes and 6,000 SNPs.

Biological pathways represent elaborate series of cascading biochemical reactions occurring within the cell, and possibly receiving external signals [7]. Pathways govern all major cellular functions, such as cell cycle, cell respiration, or apoptosis (programmed cell death). Biochemical compounds, (e.g. nucleic acids, proteins, complexes and small molecules) participating in reactions form a network of biological processes and are grouped into pathways. Reactome is an open-source, open access, manually curated and peer-reviewed pathway database (http://www.reactome.org). It visually displays structured information about the elements, enzymes, and genes (via their gene products) within many known pathways. The Reactome database was accessed in March 2013.

### Networks

Networks (or graphs) provide a means of intuitively visualizing and characterizing complex systems, and have proven to be particularly valuable in modeling biological systems. The statistical analysis of the graph properties offers a quantitative and holistic means of revealing underlying connections among vertices, as well as the emergent global properties. Networks are being used with increasing frequency to analyze large-scale systems. A network, such as PHPN, can take an extraordinarily complex system and reduce it to a relatively simple form, revealing underlying connections and important clustering details that would not be evident from studying individual or non-complex relationships among traits [8].

Formally, a network is a collection of nodes and edges connecting them. The degree, *k*, of a node is the number of edges incident upon the node, and the degree distribution, *P*(*k*) of the network, describes the fraction of nodes in the network with degree *k*. The degree distribution also characterizes global properties of the graph and how the nodes are connected to one another; for example, if they are connected at random, the nodes’ degrees are expected to be homogeneous, and the degree distribution would a uniform binomial distribution. More often in biology, networks are highly heterogeneous, with a “heavy-tailed” degree distribution, placing them in the scale-free family. This means that the degree distribution follows a power law, or exponential decay. Within the network, this translates into the presence of “hubs” – a minority of highly connected nodes. When the degree distribution of a “scale-free” network is plotted on a logarithmic scale, the resulting curve is approximately linear across the top [8]. In the case of relatively small networks, it is impossible to demonstrate the presence of a scale-free network. We can, at best, show the existence of a power-law type degree distribution, and not dismiss the scale-free hypothesis. The clustering coefficient (CC) of a network measures the degree to which nodes tend to form closely knit communities with a higher than average connectivity [9]. The CC of networks found in nature, in particular social and biological networks show a higher degree of clustering than that observed in randomized networks of identical size. The average path length of a network (APL) represents the average of the minimum number of edges separating any two vertices.

**Figure 1:**
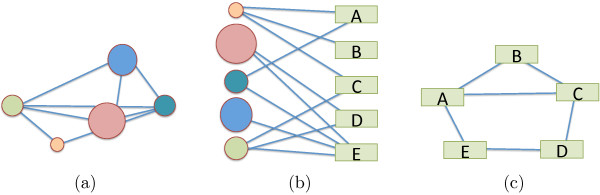
**Bipartite network schematic.** A bipartite network **(b)** made of 2 data sets the “circles”, and the “rectangles”. Projections in the “circle” space **(a)** and in “rectangle” space **(c)**.

In our study, we build a bipartite network [10], consisting of two disjoint sets of nodes. The nodes are connected in such a way that the nodes of one set will have no connections between them, but can only be connected to nodes of the other set. The use of a bipartite network is natural when dealing with two different types of data sets (Figure 1b), in our case phenotypes and pathways. Two nodes of the same type cannot connect with each other, so one node can only be connected to a node of the other data type. We used a bipartite networks to construct the relationships of our data.

From the bipartite network, one can project the data onto either of the data spaces (Figure 1a,c). In either single dataset space, the nodes are connected to one another through a vertex of the other space. By ignoring the different types of data, all network properties described above remain valid on the bipartite network (as a single data set network) and on either projection. This type of network gives us three degree-distributions, one for each projection, and one for the bipartite network. Each degree distribution shows how many links each node has. Nodes in a projection of a bipartite network are connected if they share at least one node in the other group. This gives us the ability to visualize connections within a group.

### Human disease networks

In recent years there has been a trend toward studying disease through network based analysis of various systems of connections between diseases. The result was the Human Disease Network (HDN) [1]. The nodes in the HDN represent human genetic disorders and the edges represent various connections between disorders, such as gene-gene or protein-protein interactions, to name a few. The HDN is helpful in visualizing connections among human disorders on a large scale. The underlying connections of the HDN contribute to the understanding of the basis of disorders, which in turn leads to a better comprehension of human diseases.

One study by Goh, *et al.*[1], explored the HDN built on genes shared by different diseases. Another study, which is similar in some ways to ours, by Li *et al.*[11] traced the SNPs connecting disease traits. In 2009, Silpa Suthram *et al.*[12] found that when diseases were compared by an analysis of disease-related mRNA expression data and the human protein interaction network, there were significant similarities between some diseases and between some drug treatments. In 2009, Barrenas *et al.*[4] further studied the genetic architecture of complex diseases by doing a GWAS, and found that complex disease genes are less central than the essential and monogenic disease genes in the human interactome. In the present work, we expand our study to include not only disease traits, but also behaviors and normal variations in humans, such as hair color, and explore large portions of non-coding variants in the human genome. Links between PTs are based on overlapping biological pathways (Section “Pathway-based human phenotype network”).

## Pathway-based human phenotype network

In this paper, we chose to mesh the methods and results sections, as we present multiple different algorithms (i.e. to build, filter, and identify the modules in the PHPN). Each subsection presents and applies a new method, building on the resulting network of the previous one.

### Building the PHPN

**Figure 2:**
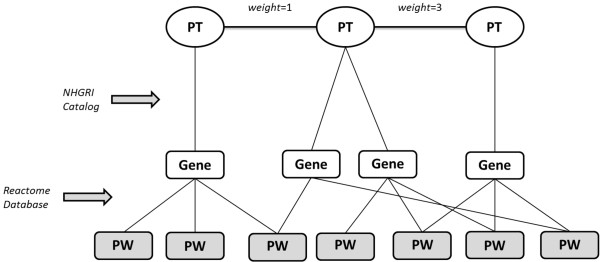
**Model of how the PHPN was built.** Phenotype-to-Gene associations were obtained using the NHGRI GWAS catalog, while gene-to-pathway associations were obtained using Reactome. Edges were drawn between phenotypes with overlapping pathways. Edge weights represent the number of overlapping pathways. PT indicates Phenotype, PW indicates Pathway.

Here we describe our method to construct a network of human phenotypes (traits and diseases) based on shared biological pathways of the associated genes. This is accomplished by linking genes to phenotypes (PTs) from hundreds of GWAS catalogue at NHGRI. Genes were further linked to pathways (PWs) using Reactome. By building these associations, we were able to link phenotypes with genes involved in the same pathways. The steps used to build the network are illustrated in Figure 2 and described as 5follow:

1. From the NHGRI catalog, extract all PTs and link them to their mapped genes. PTs with no mapped genes are omitted;

2. From Reactome, extract all genes in the database and link them to their associated pathways;

3. Match the genes associated to each phenotype to their associated pathways;

4. Connect PTs with overlapping pathways with an undirected edge, setting edge weight as the number of overlapping pathways.

We filter out isolate PTs with no connections to the rest of the network. We are only interested in PTs that have been associated with a gene, and their possible shared biology. The original NHGRI database contains over 800 PTs; by removing the isolate nodes, the PHPN contains 401 nodes connected to at least one other node.

This flexible process of building phenotype-gene-pathway associations also allowed us to examine the network from multiple configurations. Specifically, we were also able to construct a pathway network following the same logic as the HDN (Section “Human disease networks”): connecting pathways based on shared phenotypes, as well as a bipartite graph with links between PTs and pathways.

#### The bipartite network

**Figure 3:**
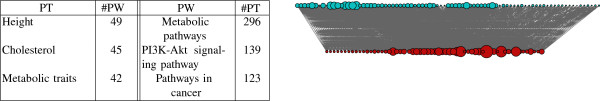
**Unfiltered bipartite phenotypes-pathways network and most connected phenotypes and pathways.** The three most connected PTs and the number of associated pathways (#PW), and the most connected PWs and the number of associated #PTs. The top (blue) row of vertices represent the phenotypes and the bottom (red) row of vertices the pathways. The vertices’ sizes are proportionate to its degree. For readability reasons, we omit vertices with degrees <24.

The Bipartite Network: The bipartite network consists of 1523 vertices (408 PTs, 1115 PWs) and over 10,000 edges, with an average degree *k*≈7 (Figure 3). We do not show the intermediate stage of the genes, as this makes the network difficult to interpret. Indeed, highly connected PT are connected to 40+ PWs, and highly connected PW, to 100–300 PTs. Height is clearly associated with most pathways, forming a major hub in the PHPN. However, it is safe to suppose that the size of the height hub represents a bias because it is recorded in most studies. It is unclear what the implications of this and other data biases are.

#### The unfiltered PHPN

**Figure 4:**
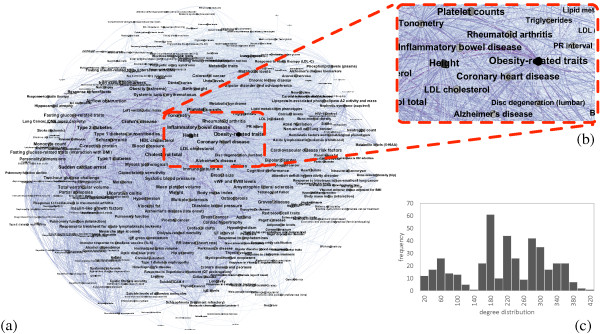
**Unfiltered pathway-based human phenotype network.(a)** The unfiltered PHPN, with nodes sizes proportionate to their degree of connectivity. **(b)** A zoom into the “obesity” region of the PHPN, including heart disease, and Alzheimer’s disease. **(c)** The degree distribution of the unfiltered PHPN.

The Unfiltered PHPN: Because we focused this study on phenotypic connections, we projected the bipartite network presented above onto the “phenotype space” (Section “Networks” and Figure 1.) The vertices in this network are only the PTs. We draw an *undirected* edge *e*_*ij*_ between two PTs *i* and *j* if they are associate to at least one common pathway. The *weightω*_*ij*_ of an edge *e*_*ij*_ is simply the number of pathways the phenotypes have in common. The result of the projection is the unfiltered Human Phenotype Network 4. It has 814 nodes and over 40,000 edges. Once the 406 isolate nodes are removed, the remaining 408 PTs and 41K edges for in a single connected component and an average degree $\bar{k}\approx 200$. Figure 4 offers a taste of how dense the network really is at this stage.

Figure 4 illustrates the sheer density of the unfiltered network and how difficult it becomes to precisely decipher the results. Even when zoomed in (Figure 4b, the network is too dense to provide any easily usable information. The degree distribution in Figure 4c does not give adequate insight into the internal structure of the network. From the results in this section, it was clear that more work had to be done on the “raw” PHPN in order for it to reveal key clinical information, both from a visual and statistical perspective. Below we describe the filtering method used and the new PHPN resulting from this filtering.

### Extracting the information backbone

Biological networks, in their raw form, are in general extremely dense. This “hairball effect” makes interpretation nearly impossible, especially from a visual perspective. Networks are, however, first and foremost visual tools. It is their relative intuitiveness and simplicity that makes them attractive for presenting data to a large audience. To make the PHPN usable and streamline our analysis, we need to extract the most significant links from the dense network: the *backbone* of the PHPN. Because of the scale-free nature of the PHPN, using a global weight (GW) threshold to eliminate edges is inappropriate. Instead, we use a multi-scale filtering algorithm outlined by Serrano *et al.*[6] to extract the HPN’s backbone. In place of a global threshold, the algorithm takes advantage of local fluctuations in edge weight to prune edges, while preserving the network’s essential structure and global properties. Specifically, we apply a disparity filter (DF) to the network; an algorithm that uses the null hypothesis that the normalized weights of the edges incident to a given node with degree *k* are produced by a random assignment from a uniform distribution. For each edge, we calculate the probability that the edge weight is compatible with the null hypothesis, which is given by:

$$\;\alpha_{\text{ij}}=1-(k-1)\int_{0}^{p_{\text{ij}}}{\left(1-x\right)}^{k-2}\text{dx}={\left(1-p_{\text{ij}}\right)}^{k-1}$$

where *k* is the degree of the node to which the edge under consideration is attached, and *p*_*ij*_ is the normalized edge weight, given by:

$$\;p_{\text{ij}}=\frac{\omega_{\text{ij}}}{s_{i}}$$

where *ω*_*ij*_ is the edge weight and *s*_*i*_ is the strength of the node under consideration (i.e. the sum of all weights of edges incident to the node). Edges are then preserved based on an imposed significance level *α*; in other words, for each edge, if *α*_*ij*_<*α*, then the edge is preserved. It should be noted that for each edge the algorithm for the DF produces two independent values *α*_*ij*_ and *α*_*ji*_ based on the two nodes connected by the edge. In order to resolve this, Serrano *et al.* propose two alternatives: under the *OR* rule, edges are preserved if (*α*_*ij*_<*α* OR *α*_*ji*_<*α*). Under the *AND* rule, both (*α*_*ij*_<*α* AND *α*_*ji*_<*α*) in order for the edge do be preserved. After experimenting with both rule types, we experimentally found the best that conserve the original network properties are obtained using the more restrictive *AND* rule. This is due to the sheer density of the unfiltered network.

**Figure 5:**
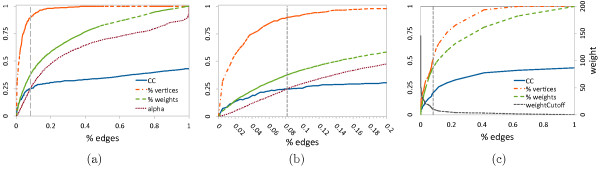
**How to choose*α*.** The networks average clustering coefficient (CC), percentage of conserved vertices (% vertices), percentage of conserved edge weights (% weights) and, in **(a)** and **(b)** the value of *α* as functions of the percentage of remaining edges (% edges). **(a)** and **(b)***α* varies in [0,1]. We chose *α*=0.25, which yields the best results, % edges =0.1, and use this value in **(c)** to identify the global weight cutoff =292. Note that curves are reported as functions of % edges, not *α*, to allow direct comparison between the DF in **(a)** and **(b)** and the GW filter in **(c)**.

Serrano *et al.*[6] have shown that the backbone analysis is more successful in extracting meaningful links from dense networks than more conventional reduction algorithms, such as global thresholds, in a variety of data sets, but not to biological data. Specifically, the algorithm reduces the number of edges while maintaining a large fraction of the nodes and weights in the unfiltered network, thus preserving many features of the network at all scales. By charting the changes in the number of edges, nodes, total weight and CC as *α* is adjusted (Figure 5), we not only demonstrate how these features are preserved in our filtered Human Phenotype Network, but also provide a rationale for which significance level cutoff to use. Indeed, Serrano *et al.*[6] have shown that these metrics give sufficient information about the network over varying values of the threshold *α* in order to ensure an adequate filtering of the network while keeping the *backbone* intact.

**Figure 6:**
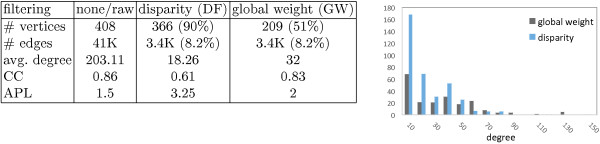
**Backbone of the HPN.** Backbone of the Pathway-based Human Phenotype Network, including the modules.

In Figure 5a and the close-up in 5b, we quantitatively identify *α*≈0.25 that conserves a CC close to that of the original network, and ∼90*%* of the PTs, ∼36*%* of the weights, and only ∼8*%* of the edges. The resulting backbone of the PHPN is presented in Figure 6.

**Figure 7:**
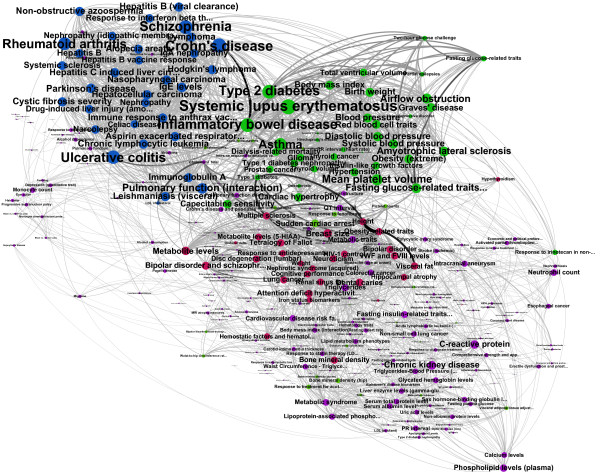
**Statistical properties.** Comparing the statistical properties of the disparity filter and the global weight filter against the unfiltered network. Percentages in parenthesis are in comparison with the unfiltered network.

To understand the advantages of the DF over a straightforward GW cutoff, we determine the cutoff value in Figure 5c that will result in a global cutoff network that also retains ∼8*%* of the edges. We compare the statistical differences between the resulting graph of these two filtering methods (Figure 7).

Results in Figure 7 clearly demonstrate the advantages of the disparity filter compared to a global weight for an identical number of edges. The DF conserves over 90% of the phenotypes versus ∼50*%* for GW. In conclusion, the backbone keeps more phenotypes than the GW filtering, for the same number of connections, making the network less dense. Moreover, the relatively low average degree, the heavy-tailed degree distribution of the PHPN backbone resulting from the DF filtering, and the high clustering coefficient and short average path length indicate an interesting module structure.

### Modules detection

In the medical literature, diseases are grouped in *disorder classes* according to an ontology of the biomedical domain [13]. In the Goh *et al.* gene-based HPN, they denote the diseases according to their disorder class [1]. Classes make “bins” in which all diseases are sorted, according to their “natural class”. Therefore, all cancers are grouped together, all cardiovascular diseases together, all gastrointestinal disorders together, and so on. We envisage two major drawbacks to this classification method: the semi-arbitrary nature of the classes, based solely on qualitative clinical observations, and not on the quantitative nature of the disorder and its underlying biology. Additionally, the manual classification is extremely tedious and subjective. We argue that in this case, we can achieve interesting results by applying a community-detecting algorithm on the filtered PHPN. This method sorts the phenotypes into classes of phenotypes with shared biology, rather than shared clinical presentation. Communities, or modules, of nodes within the network can be identified by maximizing the modularity, a measure of strength of division of a network into modules [14]. Communities are identified when a group of nodes are found to have more connections between them than would be expected by random chance, often due to some shared properties (or in our case shared biology) between the nodes in the community. The clustering coefficient (CC) measures the degree to which nodes tend to form closely knit communities with a higher than average connectivity, while a high modularity score indicates the interconnectedness, and thus the strength of the communities. The Louvain method of community detection [15] uses a greedy optimization method to maximize the modularity and determine the most favorable division of network into communities. It is a widely accepted algorithm to build communities (or modules) within a network with no expert-knowledge, although other methods, such as Infogram are widely used. Refer to Lancichinetti *et al.*’s comparative analysis [16] for more details. We run the modules detection algorithm on the backbone of the PHPN, extracting the modules detected (Figure 6).

The module detection algorithm identified 11 modules, of which 6 are part of the largest connected component, and 5 are small satellite groups of a few phenotypes. Table 1 gives the phenotypes in each group with the highest weighted degree, that is, the strongest connection to PT in the network.

**Table 1:** Modules of the PHPN

**BLUE**	**RED**	**GREEN**	**PURPLE**
**module (31)**	**module (36)**	**module (40)**	**module (201)**
Ulcerative colitis	Obesity-related traits	Inflammatory bowel disease	Triglycerides
Rheumatoid arthritis	Visceral fat	Systemic lupus erythematosus	Metabolic syndrome
Crohn’s disease	Multiple sclerosis	Type 2 diabetes	Metabolic traits
Schizophrenia	Bipolar disorder	Fasting glucose-related	Cardiovascular disease
	and schizophrenia	traits	risk factors
Immunoglobulin A	Breast size	Body mass index	Lipoprotein-associated
			phospholipase A2
			activity and mass
Parkinson’s disease	ADHD	Birth weight	C-reactive protein

The colors correspond to those in Figure 6. The number in parenthesis is the number of PT in the module.

By applying a community detection algorithm to the filtered network, we are able to classify traits and disease by quantifying their shared genetic mechanisms. This classification allows us to identify non-intuitive relationships between diseases and traits, elucidating the shared etiology for certain phenotypes.

## Clinical and biomedical implications

The appropriateness of the PHPN was assessed by examining specific edges within communities (Figure 6). Specially, we interrogated pairwise connections within the community shown in blue and asked (1) whether any constitute links between phenotypes previously known to share biological connections and (2) if they do not contain known relationships, can we understand how they may be indirectly linked based on the primary literature; thereby providing novel insights that are not only reasonable but easily visualized using our method.

### HDL cholesterol (HDL) and Alzheimer’s disease (AD)

The apolipoprotein E (*APOE*) gene is the most significantly associated gene with AD [17,18] and is also highly associated with multiple lipid traits [19-22]. The existence of an edge between HDL and AD in our network provides clear proof of principle that PHPN can detect relationships between two PTs known to be associated through a validated biological mechanism. PHPN successfully identified four common genes and six common pathways between HDL and AD (Table 2). The common pathways identified by PHPN that connect HDL and AD also support existing hypotheses about the lipid, inflammatory, and amyloid mechanisms involved in AD pathogenesis [23-26]. It is important to note that while PHPN used four common genes to detect the six common pathways between HDL and AD, these pathways harbor numerous potential candidate genes that could be used to further interrogate the genetic architecture of both AD and HDL. The promiscuous nature of the gene to pathway assignment employed by PHPN ensures that the method is robust to missingness of the genes mapped in the NHGRI catalogue.

**Table 2:** Basis of inter-node edges between HDL and AD

**Common genes**^**1**^			**Common pathways**^**2**^	
[348]	APOE		[2010]	ABC transporters
[341]	APOC1		[4520]	Adheren’s junction
[5819]	PVRL2		[5014]	Amyotrophic lateral sclerosis (ALS)
[10452]	TOMM4		[4514]	Cell adhesion molecules (CAMs)
			[4610]	Complement and coagulation cascades
			[5168]	Herpes simplex infection

^1^Associated genes shared between HDL and AD, numbers in square brackets indicate the EntrezGene ID for each specified gene.

^2^Biological pathways shared between HDL and AD, numbers in square brackets indicate Kyoto Encyclopedia of Genes and Genomes (KEGG) pathway identifiers.

### Iron status biomarkers (IB) and cognitive performance (CP)

**Figure 8:**
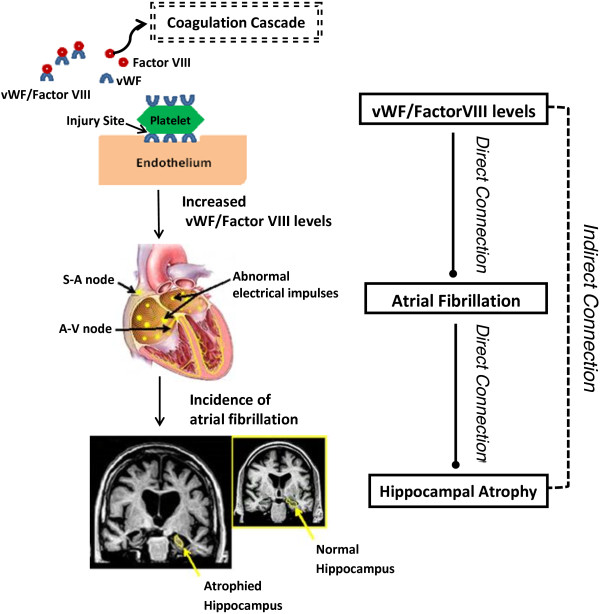
**Shared pathways.** Kyoto Encyclopedia of Genes and Genomes (KEGG) defined Biological Pathways shared between Iron Status Biomarkers and Cognitive Performance Phenotypes identified by PHPN. Note: Numbers in brackets are KEGG pathway identifiers.

There has been substantial evidence that iron is essential for dendritic growth, synaptogenesis, and myelination, and several studies indicate that early iron deficiency can lead to life-long cognitive impairment [27-29]. Importantly, upon review of the related literature, we were unable to find any single genes that were associated with both iron biomarkers and cognitive performance. However, given the clinical relevance of iron levels to neurocognitive function [30-32], we asked whether PHPN could illuminate any unknown connections between IB and CP. PHPN, as predicted, did not identify any common associated genes between IB and CP; but interestingly, the algorithm identified five enriched biological pathways that were shared between the two traits (Figure 8, and Table 3). The identification of enriched biological pathways shared between IB and CP, in the absence of any common associated genes, indicates that the connection between these two traits may be explained in part by genes located in the identified pathways that have yet to be adequately interrogated by investigators. The discovery of these shared biological pathways underscores the strength of PHPN in identifying connections between two traits that may not share any direct genic connections. This demonstrates that while PHPN utilizes the information gained from GWAS studies to identify phenotypic connections, even in the absence of explicit genic connections it is still able to identify important relationships between PTs.

**Table 3:** Kyoto Encyclopedia of Genes and Genomes (KEGG) defined biological pathways shared between iron status biomarkers and cognitive performance phenotypes

**Iron biomarker pathways**^**1**^		**Cognitive performance pathways**^**2**^		**Common pathways**^**3**^	
[4142]	Lysosome	[5143]	African trypanosomiasis	[1100]	Metabolic pathways
[1100]	Metabolic pathways	[5034]	Alcoholism	[3060]	Protein export
[4978]	Mineral absorption	[5010]	Alzheimers disease	[4066]	HIF-1 signaling
[3060]	Protein export	[5146]	Amoebiasis	[4151]	PI3K-Akt signaling
[4060]	Cytokine-cytokine receptor interaction	[5142]	Chagas disease (American trypanosomiasis)	[4080]	Neuroactive ligand-receptor interaction
[531]	Glycosaminoglycan degradation	[4961]	Endocrine and other factor-regulated calcium reabsorption		
[4066]	HIF-1 signaling	[650]	Butanoate metabolism		
[4080]	Neuroactive ligand-receptor interaction	[4020]	Calcium signaling		
[4151]	PI3K-Akt signaling	[5031]	Amphetamine addiction		
[4630]	Jak-STAT signaling	[4062]	Chemokine signaling		
[4120]	Ubiquitin mediated proteolysis	[280]	Valine leucine and isoleucine degradation		
		[4713]	Circadian entrainment		
		[5030]	Cocaine addiction		
		[4060]	Cytokine-cytokine receptor interaction		
		[4728]	Dopaminergic synapse		
		[5014]	Amyotrophic lateral sclerosis (ALS)		
		[4144]	Endocytosis		
		[5169]	Epstein-Barr virus infection		
		[71]	Fatty acid metabolism		
		[4510]	Focal adhesion		
		[4727]	GABAergic synapse		
		[4540]	Gap junction		
		[4971]	Gastric acid secretion		
		[4724]	Glutamatergic synapse		
		[4912]	GnRH signaling		
		[4066]	HIF-1 signaling		
		[5016]	Huntingtons disease		
		[562]	Inositol phosphate metabolism		
		[4730]	Long-term depression		
		[4720]	Long-term potentiation		
		[310]	Lysine degradation		
		[4916]	Melanogenesis		
		[1100]	Metabolic pathways		
		[5032]	Morphine addiction		
		[4080]	Neuroactive ligand-receptor interaction		
		[5033]	Nicotine addiction		
		[4330]	Notch signaling		
		[670]	One carbon pool by folate		
		[4151]	PI3K-Akt signaling		
		[3320]	PPAR signaling		
		[4972]	Pancreatic secretion		
		[4146]	Peroxisome		
		[4070]	Phosphatidylinositol signaling system		
		[640]	Propanoate metabolism		
		[3060]	Protein export		
		[4723]	Retrograde endocannabinoid signaling		
		[5323]	Rheumatoid arthritis		
		[4970]	Salivary secretion		
		[4726]	Serotonergic synapse		
		[4721]	Synaptic vesicle cycle		
		[5322]	Systemic lupus erythematosus		
		[5202]	Transcriptional misregulation in cancer		
		[380]	Tryptophan metabolism		
		[4725]	Cholinergic synapse		
		[4270]	Vascular smooth muscle contraction		
		[4310]	Wnt signaling		
		[410]	beta-Alanine metabolism		

Note: Numbers in square brackets “[ ]” represent KEGG database identifiers for indicated biological pathways.

^1^KEGG Pathways assigned to the Iron Status Biomarker Pathway.

^2^KEGG Pathways assigned to the Cognitive Performance Pathway.

^3^KEGG Pathways that are shared between the iron status biomarker and cognitive performance pathways.

### von Willebrand factor and FVIII levels (vWF) and hippocampal atrophy (HA)

**Figure 9:**
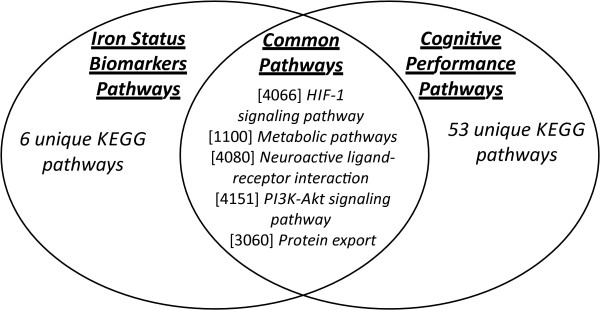
**Diseases connection mechanism.** Proposed Mechanism of indirect connection between von Willebrand Factor/Factor VIII and Hippocampal Atrophy via Atrial fibrillation. *Note*: Underlying MRI image was obtained and adapted from: http://www.healthjolt.com/features/heflin/ alzheimers-disease/. Underlying heart image was obtained and adapted from: http://caifl.com/arrhythmia-information/atrial-fibrillation/ copyright.

Two traits that were connected in the PHPN but did not share any common associated genes or any clear-cut biological relationship were vWF and HA. vWF promotes platelet adhesion to subendothelial tissues at the site of vascular injury and is the carrier protein for coagulation factor VIII (FVIII); FVIII acts as a co-factor in the coagulation cascade accelerating the activation of factor X by factor IX [33,34]. Together, vWF and FVIII levels are important hemostatic factors involved in the pathophysiology of various blood [35,36] and cardiovascular [37,38] conditions. Additionally, circulating vWF is used as a biomarker for inflammation [39]. Hippocampal atrophy (HA) is characterized by decreased hippocampal volume. Because the hippocampus is the region of the brain that is essential for memory formation, abnormalities in this region have been seen in various neurodegenerative disorders such as dementia and AD [40,41]. PHPN identified a connection between vWF and HA with the unifying factor being a single shared pathway (Table 4). Because the relationship between these two PTs was not expected, we examined possible biological connections between the two via literature review. Upon review, we discovered a recently published study that interrogated inflammatory biomarkers for association with hippocampal volume; it is important to note however that the biomarkers assessed in this study did not include vWF [42]. Further research revealed strong associations between atrial fibrillation and both phenotypes; increased levels of vWF associate with incidence of atrial fibrillation [43], and incidence of atrial fibrillation associates with increased hippocampal atrophy [44,45] (Figure 9).

**Table 4:** Enriched biological pathways assigned to vWF, HA, and AF by PHPN

**Von Willebrand factor/**		**Atrial fibrillation (AF) pathways**^**1**^		**Hippocampal atrophy (HA)**^**1**^	
**factor VIII pathways**^**1**^				**pathways**	
[5412]	Arrhythmogenic right ventricular	***[1100]***	***Metabolic pathways***	[4360]	Axon guidance
[4662]	B cell receptor signaling pathway	[670]	One carbon pool by folate	[4623]	Cytosolic DNA-sensing pathway
[4514]	Cell adhesion molecules (CAMs)			[531]	Glycosaminoglycan degradation
[4062]	Chemokine signaling pathway			[4142]	Lysosome
[4664]	Fc epsilon RI signaling pathway			***[1100]***	***Metabolic pathways***
***[1100]***	***Metabolic pathways***			[510]	N-Glycan biosynthesis
[4666]	Fc gamma R-mediated phagocytosis			[4151]	PI3K-Akt signaling pathway
[4510]	Focal adhesion			[4141]	Protein processing in endoplasmic reticulum
[4670]	Leukocyte trans endothelial migration			[4530]	Tight junction
[4650]	Natural killer cell mediated cytotoxicity				
[4810]	Regulation of actin cytoskeleton				
[4660]	T cell receptor signaling pathway				
[4742]	Taste transduction				

^1^Pathways listed are identified enriched pathways for indicated phenotypes; numbers in square brackets indicate the Kyoto Encyclopedia of Genes and Genomes (KEGG) identifiers for specified pathways.

^2^Common biological pathway is indicated in bold.

Through this analysis, PHPN exposed atrial fibrillation phenotype as a key connector between VWF and HA even though none of these three PTs share any common genetic risk factors, as reported in the GWAS catalogue, but all three phenotypes shared a common biological pathway (Table 4). Therefore, the PHPN was able to identify a possible, and plausible, indirect relationship between vWF and HA through the unifying, but independent, phenotype of atrial fibrillation Thus, PHPN provides a novel means to identify inter-relationships between hemostatic, cardiovascular, and neurological conditions that may otherwise have gone unnoticed. It is also interesting to note that the single overlapping pathway between vWF and HA, the KEGG aggregate Metabolic Pathway ([1100]), is an comprehensive pathway consisting of all the metabolic pathways contained in the KEGG database, comprising approximately 10% of the human genome (≈2000 genes). Of the pathways interrogated by PHPN for this study 142 were linked through this “umbrella” pathway. Further investigation of the overall network revealed that of these 142 unique phenotypes, only three shared only the Metabolic pathway in common; vWF, HA, and AF, although AF was located in a different module which consisted mainly of cardiovascular diseases. The identification of plausible underlying biology between two phenotypes who share only this pathway in common suggest that PHPN displays a certain amount of robustness to ambiguous pathway definition by KEGG.

## Discussion

PHPN provides a means of integrating the accumulating wealth of genomic and phenotypic data and computationally identifies significant links between traits, attributes and diseases. This model has tremendous potential as a clinical tool in identifying risk factors for certain diseases, or common drug targets. By constructing a network based on pathways, we were able to associate phenotypes based on the shared biological processes involving common genetic components and pleiotropic effects. Our network of human traits based on ∼ 2,300 genes, ∼ 1,200 biological pathways and 800+ phenotypes is more comprehensive than that of previous studies. We combine GWAS data, which associates PT to genes, with the data from Reactome, which links genes to pathways. We extract the backbone of the PHPN using the disparity filter, retaining the significant connection. Our statistical analysis of the network properties places the PHPN in the scale-free family, showing once more how ubiquitous network structures with heavy-tailed degree distributions really are in biological, social, and natural networks. The automatic classification of phenotypes into “phenotype classes”, using the network’s topological modularity and a standard community detection algorithm, showed very promising results. Indeed, in contrast to what was achieved in previous studies and manual classification, we are able to highlight modules with phenotypes with potentially interesting shared biology, not by arbitrary disease types. Despite its apparent simplicity, PHPN is an adaptable network algorithm that can elucidate both intuitive and previously undiscovered biological connections between PTs, deftly characterizing the shared genetic mechanisms in the former and identifying unifying genetic traits in the latter. The ability to recognize biological connections, quantified by shared genes and their associated biological pathways, between seemingly disparate phenotypes provides researchers with a unique view of the pleiotropic biological environment that underlies the human condition. Discovering additional, novel, connections between phenotypes known to share certain biological traits provides additional information that could be exploited in future hypothesis based studies. Recognizing the connections between different traits/phenotypes is an integral first step in understanding the dynamic, and highly inter-related, genetic architecture underlying most complex disease; once these connections are illuminated they may provide the necessary framework for the generation of novel and innovative therapeutic interventions. For future work, we are interested in integrating more datasets on gene interactions into our network, such as SNPs and protein-protein interactions. Furthermore, we are currently working on three angles, (1) comparing the HPN to the HDN, and other previous work on phenotype networks, (2) running statistical significance tests, such as data set randomization, and finally (3) on refining our statistical methods, comparing algorithms for pruning our network and identifying communities that may produce optimal results in extracting the significant interactions in the PHPN.

## Competing interests

The authors declare that they have no competing interests.

## Authors’ contributions

CD, HPN experimental design, network analysis, manuscript redaction. MJW, Clinical and Biomedical analysis and section redaction. BEG, HPN statistical network analysis, visualization, background sections redactions. DNL, HPN methods implementation. SW, Clinical and Biomedical analysis. JHM, experimental design, network analysis. All authors read and approved the final manuscript.
